# The International Collaboration on Air Pollution and Pregnancy Outcomes: Initial Results

**DOI:** 10.1289/ehp.1002725

**Published:** 2011-02-09

**Authors:** Jennifer D. Parker, David Q. Rich, Svetlana V. Glinianaia, Jong Han Leem, Daniel Wartenberg, Michelle L. Bell, Matteo Bonzini, Michael Brauer, Lyndsey Darrow, Ulrike Gehring, Nelson Gouveia, Paolo Grillo, Eunhee Ha, Edith H. van den Hooven, Bin Jalaludin, Bill M. Jesdale, Johanna Lepeule, Rachel Morello-Frosch, Geoffrey G. Morgan, Rémy Slama, Frank H. Pierik, Angela Cecilia Pesatori, Sheela Sathyanarayana, Juhee Seo, Matthew Strickland, Lillian Tamburic, Tracey J. Woodruff

**Affiliations:** 1 National Center for Health Statistics, Centers for Disease Control and Prevention, Hyattsville, Maryland, USA; 2 Department of Community and Preventive Medicine, University of Rochester School of Medicine and Dentistry,Rochester, New York, USA; 3 Institute of Health and Society, Newcastle University, Newcastle upon Tyne, England, United Kingdom; 4 Department of Occupational and Environmental Medicine, Inha University, Incheon, Republic of Korea; 5 UMDNJ-Robert Wood Johnson Medical School, Piscataway, New Jersey, USA; 6 Yale University, School of Forestry and Environmental Studies, New Haven, Connecticut, USA; 7 Department of Experimental Medicine, University of Insubria, Varese, Italy; 8 University of British Columbia, Department of Medicine, Vancouver, British Columbia, Canada; 9 Department of Environmental Health, Emory University, Atlanta, Georgia, USA; 10 Institute for Risk Assessment Sciences, Utrecht University, Utrecht, the Netherlands; 11 Department of Preventive Medicine, School of Medicine of the University of São Paulo, São Paulo, Brasil; 12 Epidemiology Unite, “Fondazione IRCCS Ca’Granda—Ospedale Maggiore Policlinico,” Milan, Italy; 13 Department of Preventive Medicine, Ewha Womans University, Seoul, Republic of Korea; 14 Generation R Study Group, Erasmus Medical Center, Rotterdam, the Netherlands; 15 Department of Urban Environment, Netherlands Organisation for Applied Scientific Research (TNO), Delft, the Netherlands; 16 Centre for Research, Evidence Management and Surveillance, Sydney South West Area Health Service, and School of Public Health and Community Medicine, University of New South Wales, Sydney, Australia; 17 Department of Environmental Science, Policy and Management, University of California–Berkeley, Berkeley, California, USA; 18 INSERM, Team of Environmental Epidemiology applied to Reproduction and Respiratory Health, U823, Institut Albert Bonniot, Grenoble, France.; 19 University J. Fourier Grenoble, Grenoble, France; 20 School of Public Health, University of California–Berkeley, Berkeley, California, USA; 21 North Coast Area Health Service, Lismore, New South Wales, Australia; 22 University Centre for Rural Health–North Coast, University of Sydney, Sydney, New South Wales, Australia; 23 Department of Occupational and Environmental Health, Università di Milano, Milan, Italy; 24 Seattle Children’s Research Institute, University of Washington, Seattle, Washington, USA; 25 University of British Columbia, Centre for Health Services and Policy Research, Vancouver, British Columbia, Canada; 26 Center for Reproductive Health and the Environment. University of California–San Francisco, San Francisco, California, USA

**Keywords:** air pollution, birth weight, ICAPPO, low birth weight, particulate matter, pregnancy

## Abstract

Background: The findings of prior studies of air pollution effects on adverse birth outcomes are difficult to synthesize because of differences in study design.

Objectives: The International Collaboration on Air Pollution and Pregnancy Outcomes was formed to understand how differences in research methods contribute to variations in findings. We initiated a feasibility study to *a*) assess the ability of geographically diverse research groups to analyze their data sets using a common protocol and *b*) perform location-specific analyses of air pollution effects on birth weight using a standardized statistical approach.

Methods: Fourteen research groups from nine countries participated. We developed a protocol to estimate odds ratios (ORs) for the association between particulate matter ≤ 10 μm in aerodynamic diameter (PM_10_) and low birth weight (LBW) among term births, adjusted first for socioeconomic status (SES) and second for additional location-specific variables.

Results: Among locations with data for the PM_10_ analysis, ORs estimating the relative risk of term LBW associated with a 10-μg/m^3^ increase in average PM_10_ concentration during pregnancy, adjusted for SES, ranged from 0.63 [95% confidence interval (CI), 0.30–1.35] for the Netherlands to 1.15 (95% CI, 0.61–2.18) for Vancouver, with six research groups reporting statistically significant adverse associations. We found evidence of statistically significant heterogeneity in estimated effects among locations.

Conclusions: Variability in PM_10_–LBW relationships among study locations remained despite use of a common statistical approach. A more detailed meta-analysis and use of more complex protocols for future analysis may uncover reasons for heterogeneity across locations. However, our findings confirm the potential for a diverse group of researchers to analyze their data in a standardized way to improve understanding of air pollution effects on birth outcomes.

Evidence that poor air quality can adversely affect birth outcomes is increasing. A small number of review articles have summarized existing studies and concluded that there is likely an adverse effect of air pollution on pregnancy outcome (Glinianaia et al. 2004; Ritz and Wilhelm 2008; Šrám et al. 2005). However, estimated associations between these outcomes and air pollutant exposures over the whole pregnancy and during specific time windows (e.g., trimester of pregnancy) have been inconsistent, making definitive conclusions difficult (Glinianaia et al. 2004; Slama et al. 2008; Woodruff et al. 2009).

Comparisons of findings across different geographic locations are hindered, in part, by differences in research designs. Although most published studies have reported adverse pregnancy outcomes in association with prenatal exposure to air pollution, inconsistent findings reported by some studies prompted a series of workshops to discuss this relatively new area of investigation (Slama et al. 2008; Woodruff et al. 2009) and the formation of the International Collaboration on Air Pollution and Pregnancy Outcomes (ICAPPO) (Woodruff et al. 2010). The primary objective of ICAPPO is to understand how differences in research design and methods contribute to variations in findings.

As part of this effort, a feasibility study was developed to determine whether it would be possible to use a common protocol to reanalyze existing data sets that were created to answer similar but not identical research questions. A workshop was held in Dublin (25–29 August 2009) to share and discuss the initial results of the feasibility study. In this report, we describe the common research protocol and participating studies. Throughout this article, study results from each research group are referred to by name [e.g., EDEN study (Etude des Déterminants pré et post natals du développement et de la santé de l’Enfant)] if available, otherwise by location (e.g., Seattle study). Additionally, we present estimated odds ratios (ORs) for the association between low birth weight (LBW) among term births and exposure to ambient particulate matter with an aerodynamic diameter ≤ 10 μm (PM_10_) during pregnancy.

## Methods

Through discussion with the larger group of ICAPPO participants and detailed planning by a smaller group (J.D.P., D.Q.R., S.V.G., J.H.L.), a protocol for the feasibility study was developed, agreed upon, and distributed to a geographically diverse group of researchers. To maximize the number of participating groups, we deliberately simplified the protocol by restricting the primary statistical analysis to one outcome (LBW in term births) and the air pollution exposure (PM_10_) available for the largest number locations (Woodruff et al. 2010).

*Cohort restrictions.* We limited the study to live-born, singleton, term (37–42 complete weeks of gestation) infants with known birth weight, maternal education [or another measure of socioeconomic status (SES)], dates of birth and conception (often based on last menstrual period), and ambient PM concentrations, as described below, during pregnancy. The primary outcome was term LBW, defined as birth weight < 2,500 g.

*Air pollution exposure.* The primary exposure variable was the ambient concentration of PM_10_ averaged over the entire pregnancy. PM_10_ concentrations were assigned to each subject using the approach employed by each research group in their original work. Although we focused on PM_10_, investigators also were encouraged to provide results for fine PM [≤ 2.5 μm in aerodynamic diameter (PM_2.5_)] if available. Studies without PM_10_ data provided effect estimates for PM_2.5_ or black smoke exposures during pregnancy.

Black smoke approximates PM_4_ (< 4 µm in diameter) (Muir and Laxen 1995); results for black smoke are presented alongside the PM_10_ results for the PAMPER (Particulate Matter and Perinatal Events Research) study (Newcastle upon Tyne, UK). The methods for modeling the PAMPER black smoke exposures are described elsewhere (Fanshawe et al. 2008).

*Socioeconomic status.* ICAPPO participants identified SES as a potentially important control variable when assessing pollution and birth outcomes (Slama et al. 2008; Woodruff et al. 2009) and agreed to use maternal education as the primary measure of SES in the feasibility study. Maternal education is commonly used as an SES measure in perinatal studies and has been shown to be related, albeit imperfectly, with other measures of SES (Kaufman et al. 2008; Parker et al. 1994; Pickett et al. 2002). If maternal education was unavailable, using different individual or area-level SES measures was allowed. Because the collection and meaning of maternal education for these studies differ among the study locations, its form as an analytic covariate differed among the study locations.

*Other covariates.* Participants also were encouraged to provide estimates adjusted for additional covariates as described below. Although additional variables make comparisons of results across locations more challenging, they allowed us to examine how additional adjustments specific to each location might influence estimates reported by each study.

*Primary statistical analysis.* We used logistic regression, with term LBW as the dependent variable and PM_10_ as a continuous explanatory variable; black smoke was used in the PAMPER study, as described above. Results are reported as ORs per 10-μg/m^3^ increase in average concentration during pregnancy to facilitate synthesis of results. Results from two models were examined: Model 1 covariates were PM_10_ and study-specific maternal education or other SES measure; model 2 covariates were PM_10_, maternal education or other SES measure, plus other study location–specific covariates as described above.

*Secondary statistical analyses.* For these analyses, we suggested modeling continuous term birth weight as an outcome (using linear regression) and/or using PM_2.5_ as an exposure measure. In addition, results from models describing associations after controlling for different SES measures were contributed. Secondary analyses were encouraged but not required for participation, so results of secondary analyses were not reported by all investigators.

Although full meta-analyses were not performed, in our examination of results, initial tests of homogeneity across study locations were conducted using fixed-effects models (Sterne et al. 2001). In these tests, the null hypothesis of homogeneity was rejected with *p*-values < 0.05.

## Results

*Locations.* Fourteen research groups from nine countries participated (Table 1). Of these, six reported results for PM_10_ only, six for both PM_10_ and PM_2.5_, one for PM_2.5_ only (Seattle study), and one for black smoke only (PAMPER study). Most data were from the late 1990s to the mid-2000s. However, the PAMPER study comprised births from 1962 through 1992. The number of eligible births ranged from slightly > 1,000 in the EDEN study, Nancy and Poitiers, France] to > 1 million in the California study, although there was some variability within studies depending on the exposure measure and covariates. The percentage of LBW among term births ranged from 1.15% in the PIAMA (Prevention and Incidence of Asthma and Mite Allergy) study (Netherlands) to 3.77% in the São Paulo study (Table 1).

**Table 1:** Birth years, number of births, percent term LBW, and measure of SES used in model 1 (adjusted for SES only), by study.

Table 1. Birth years, number of births, percent term LBW, and measure of SES used in model 1 (adjusted for SES only), by study.										
				No. of births*b*		Percent term LBW		SES measure used in model 1 of feasibility study		
Study and location*a*		Birth years						Measure		Descriptive statistics
Atlanta, Georgia, USA (Darrow et al. 2009a, 2009b)		1996–2004		325,221		2.62		Attained maternal education		Years: 19.8% < 12, 24.7% 12, 55.5% > 12
California, USA (Morello-Frosch et al. 2010)		1996–2006		1,714,509		2.43		Attained maternal education*c*		Years: 31.5% < 12, 28.0% 12, 40.5% > 12
Connecticut and Massachusetts, USA (Bell et al. 2007, 2008)		1999–2002		173,042		2.16		Attained maternal education		Mean ± SD, 13.6 ± 2.6 years
EDEN, Poitiers and Nancy, France (Lepeule et al. 2010)		2003–2006		1,233		2.11		Age at completion of education		Years: 17.7% < 19, 61.7% 19–24, 20.6% > 24
Lombardy, Italy (Pesatori et al. 2008)		2004–2006		213,542		2.71		Attained maternal education		Degree: 33.3% < high school, 45.8% high school, 3.6% bachelor, 17.6% graduate
PAMPER, Newcastle upon Tyne, UK (Glinianaia et al. 2008; Pearce et al. 2010)		1962–1992		81,953		3.19		Area-level indicator: Townsend Deprivation Score*d*		Quintile cut-points: –1.2, 2.4, 4.7, 6.6
New Jersey, USA (Rich et al. 2009)		1999–2003		87,281		2.75		Attained maternal education		Years: 20.6% < 12, 36.5% 12, 42.9% > 12
PIAMA, the Netherlands (Gehring et al. 2011)		1996–1997		3,471		1.15		Attained maternal education		Degree: 22.8% low, 41.6% medium, 35.6% high
Generation R, Rotterdam, the Netherlands (van den Hooven et al. 2009)		2002–2006		7,296		2.26		Attained maternal education		Degree: 10.9% none/low, 44.7% secondary, 44.3% higher
São Paulo, Brazil (Gouveia et al. 2004)		2005		158,791		3.77		Attained maternal education		Years: 29.3% < 7, 50.7% 8–11, 19.9% > 11
Seoul, Republic of Korea (Ha et al. 2004)		1998–2000		372,319		1.45		Attained maternal education		Degree: 4.1% < high school, 52.7% high school, 43.2% ≤ bachelor
Seattle, Washington, USA (Sathyanarayana S, Karr C, unpublished data)		1998–2005		301,880		1.56		Attained maternal education*c*		Years: 12.8% < 12, 26.1% 12, 60.0% > 12
Sydney, Australia (Jalaludin et al. 2007)		1998–2004		279,015		1.62		Area-level indicator: Index of Relative Socioeconomic Disadvantage*e*		Quartile cut-points: ≤ 945.1, 1010.7, 1072.7
Vancouver, British Columbia, Canada (Brauer et al. 2008)		1999–2002		66,467		1.35		Area level indicator: percentage of women with postsecondary education		Quartile cut-points: 28.8, 36.3, 44.1
**a**Data sets have been used for other studies, although not necessarily studies of PM_10_ or term LBW; cited analyses sometimes used different versions of the data. **b**Births used in model 1: singleton, term infants with known birth weight, maternal SES, gestational age, and ambient PM_10_ or black smoke concentrations. **c**Collection of maternal education changed during the study period. **d**The Townsend Deprivation Score is an area-based measure of material deprivation (Townsend et al. 1988), calculated for each enumeration district (~ 200 households) based on 1971, 1981, and 1991 census data. **e**The Australian Bureau of Statistics (2001) Index of Relative Socio-economic Disadvantage uses a range of census factors and is assigned to each census collection district (~ 200 households).										

By design, data sets used in the feasibility study have been used for previous studies of pollution and pregnancy outcomes or are intended for such use. However, these are not necessarily studies of PM_10_ or term LBW, and previously published results may have been based on earlier versions of study data sets (Bell et al. 2007, 2008; Brauer et al. 2008; Darrow et al. 2009a, 2009b; Gehring et al. 2011; Glinianaia et al. 2008; Gouveia et al. 2004; Ha et al. 2004; Jalaludin et al. 2007; Lepeule et al. 2010; Mannes et al. 2005; Pearce et al. 2010; Pesatori et al. 2008; Rich et al. 2009; Slama et al. 2009; van den Hooven et al. 2009).

*PM concentration estimation.* PM concentration estimates and estimation methods differed among the studies (Table 2). Some research groups relied on temporal variability in PM to estimate effects, where exposure was calculated by averaging all measurements over the entire study area for the pregnancy interval; for these studies, exposure estimates differed for pregnancies occurring at different times, but not by maternal residence, within the study area. Other studies estimated effects based on both temporal and spatial PM contrasts, where estimates were calculated for multiple geographic administrative units or at each maternal address; in these studies, exposures differed both by maternal address and by timing of the pregnancies within the study period. Most research groups (11 of 14; 79%) used routinely collected monitoring network data to estimate exposures (Table 2), although its use differs among studies [e.g., averages over geographic areas; nearest monitor measurement, or inverse distance-weighted (IDW) averages from multiple monitors, from residence].

**Table 2:** PM_10_ distribution, method of exposure estimation, area, and source of exposure variability, by study.

Table 2. PM_10_ distribution, method of exposure estimation, area, and source of exposure variability, by study.												
		PM_10_ distribution (μg/m^3^)								Approximate area*a* (km^2^)		
Study		Median		25th percentile		75th percentile		Method of exposure estimation				Exposure contrast*b*
Atlanta		23.5		22.3		25.4		Monitoring network; population-weighted spatial average over city (Ivy et al. 2008)		4,538		Temporal
California		28.9		22.6		38.7		Monitoring network; nearest monitor within 10 km of residence		423,970*a*		Spatial and temporal
Connecticut and Massachusetts		22.0		18.1		25.5		Monitoring network; spatial average over county of residence		41,692		Spatial and temporal
EDEN		19.0		18		21		Monitoring network; nearest monitor within 20 km of residence		480		Spatial and temporal
Lombardy		49		44		54		Monitoring network; average of monitoring stations located in nine regional areas (Baccarelli et al. 2007)		23,865		Spatial and temporal
PAMPER*c*		(PM_10_ not available)						Spatial-temporal model for black smoke (Fanshawe et al. 2008)		63		Spatial and temporal
New Jersey		28.0		24.8		31.7		Monitoring network; nearest monitor within 10 km of residence		22,592*a*		Spatial and temporal
PIAMA		40.5		36.7		43.4		LUR model (Gehring et al. 2011) with temporal adjustment using air monitoring network data*d*		12,000		Spatial and temporal
Generation R		32.8		32.2		33.3		Dispersion model (Wesseling et al. 2002)		150		Spatial
São Paulo		40.3		39.2		42.1		Monitoring network; average from 14 monitors throughout city		1,500		Temporal
Seattle*e*		(PM_10_ not available)						Monitoring network; population-weighted spatial average of PM_2.5_ for monitors within 20 km of residence (Ivy et al. 2008)		17,800		Spatial and temporal
Seoul		66.45		59.63		69.72		Monitoring network; average from 27 monitors throughout city		605		Spatial and temporal
Sydney		16.50		12.8		21.0		Monitoring network; average from eight monitors throughout city		12,145		Temporal
Vancouver		12.5		11.7		13.1		Monitoring network; inverse distance weighting of up to three monitors within 50 km of residence*f*		3,300		Spatial and temporal
**a**Approximate geographic area in which mothers reside; in California and New Jersey, the geographic area includes maternal addresses too far from a PM_10_ or PM_2.5_ monitoring site to be included in the study. **b**Temporal contrast is used to describe studies where exposure estimates differ among mothers based on the timing of their pregnancy; spatial contrast is used to describe studies where exposure estimates differ among mothers based on their residence. **c**Only black smoke available (black smoke is a historic measure of airborne PM, ~ PM_4_, shown to be a reasonable predictor of daily average PM_10_) (Muir and Laxen 1995). **d**PM_10_ estimated from PM_2.5_ LUR model results. **e**Only PM_2.5_ available. **f**PM_2.5_ exposure also derived from LUR (see “PM concentration estimation”).												

Two research groups used models to estimate PM_10_ exposure (Table 2), although modeling methods differed. The Generation R study (Rotterdam, the Netherlands) used dispersion modeling (combination of monitoring data with modeling techniques) (Wesseling et al. 2002), whereas the PIAMA study (Netherlands) used temporally adjusted land use regression (LUR) (Gehring et al. 2011) and estimated residential PM_10_ from modeled PM_2.5_ concentration (Cyrys et al. 2003). PAMPER used modeled estimates, as described above; the median modeled black smoke concentration in the PAMPER data set was 32.8 μg/m^3^ with an interquartile range of 17.1–104.9, reflecting, in part, the long time spanned. The Vancouver study used monitoring network data for PM_10_ but used both LUR models and monitoring network data (IDW) to estimate PM_2.5_ exposures (Brauer et al. 2008); results for both Vancouver PM_2.5_ estimates are shown below.

*Socioeconomic status.* Eleven of the 14 research groups used maternal education as the indicator of SES for model 1 (Table 1). However, the maternal education measure varied in form and meaning across studies. Three studies relied on contextual information based on neighborhood characteristics to define maternal SES for model 1 of the primary analysis (Table 1). Some research groups included additional individual level socioeconomic measures for model 2 and in secondary analyses [see Supplemental Material, Table 1 (doi:10.1289/ehp.1002725)]. For example, paternal occupation was used in the Lombardy study. The California study added area-level socioeconomic measures. Similarly, the Vancouver study added an additional area-level income variable. Some research groups included individual-level characteristics that may correlate with SES: maternal age, race, ethnicity, indigenous status, and country of birth.

**Figure 1:**
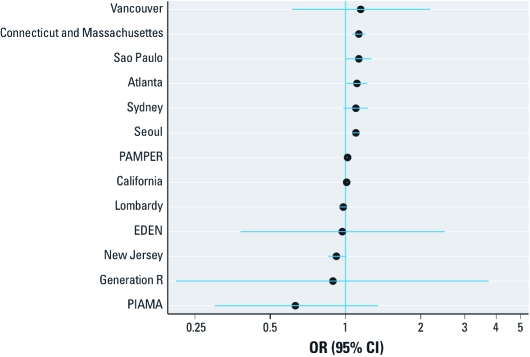
ORs (95% CIs) for LBW among term births in association with a 10‑μg/m^3^ increase in estimated average PM_10_, or black smoke (PAMPER), concentration during the entire pregnancy, adjusted for SES (model 1), by study.

*Birth weight.* Figure 1 shows the relative odds of term LBW per 10-μg/m^3^ increase in mean PM_10_ concentration during pregnancy, adjusted for SES (model 1) by location. Associations differed among study locations (*p*-value from test for heterogeneity < 0.001). Six studies indicated a statistically significant positive (adverse) association (Atlanta, California, Connecticut and Massachusetts, PAMPER, São Paulo, and Seoul), whereas the Sydney and Vancouver studies indicated an adverse, albeit not significant, association (Figure 1). Little or no association was reported by seven studies; no research group reported significant inverse (protective) associations.

**Figure 2:**
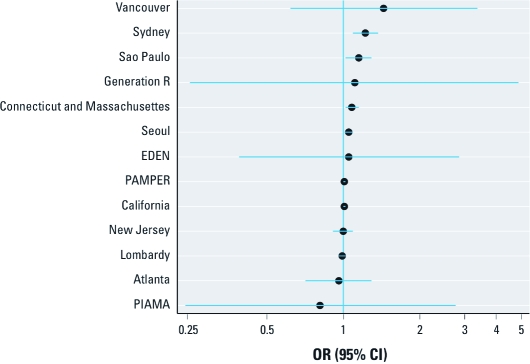
ORs (95% CIs) for LBW among term births in association with a 10‑μg/m^3^ increase in estimated average PM_10_, or black smoke (PAMPER), concentration during the entire pregnancy, adjusted for SES and study-specific variables (model 2), by study.

Figure 2 shows estimated ORs from model 2 [models fitted with additional covariates; see Supplemental Material, Table 1 (doi:10.1289/ehp.1002725)]. Additional covariates varied among studies and included maternal age and transformations of age, parity, antenatal visits, country of birth, sex, maternal smoking, maternal alcohol, maternal hypertension, maternal diabetes, season of conception, year of birth, marital status, race/ethnicity, indigenous status, gestational age, and contextual measures of SES. About half of model 2 ORs suggest slightly stronger associations between air pollution and term LBW compared with model 1 ORs, whereas other model 2 ORs were either very similar or attenuated compared with model 1 [for a direct comparison of estimates, see Supplemental Material, Table 2 (doi:10.1289/ehp.1002725). Associations differed among study locations (*p*-value from test for heterogeneity < 0.05).

**Figure 3:**
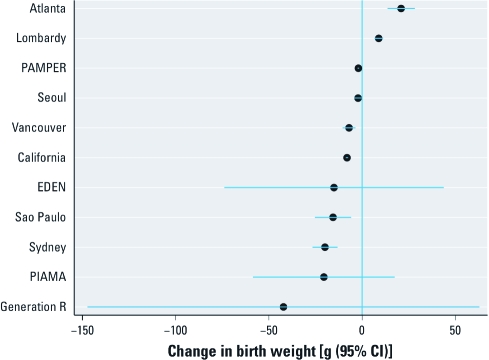
Change in mean birth weight (95% CIs) among term births in association with a 10‑μg/m^3^ increase in estimated average PM_10_, or black smoke (PAMPER), concentration during the entire pregnancy, adjusted for SES, by study.

Figure 3 shows changes in mean term birth weight associated with each 10-μg/m^3^ increase in PM_10_ for the 11 locations reporting continuous birth weight results. The mean estimated change ranged from a 42.2-g decrease (Generation R) to an increase of about 20 g (the Atlanta study), with most estimates (9 of 11) indicating a 2- to 20-g lower birth weight associated with each 10-μg/m^3^ increase in PM_10_ exposure. Of the 11 studies, six reported a statistically significant adverse effect of PM_10_, whereas two (the Atlanta and Lombardy studies) indicated a significant protective effect. These associations differed among study locations (*p*-value from test for heterogeneity < 0.001). After controlling for study-specific factors, model coefficients often, although not always, suggested larger decreases in birth weight with increases in PM_10_ [see Supplemental Material, Table 3 (doi:10.1289/ehp.1002725)]. In the Atlanta study, the estimate changed from an apparent mean increase of 20 g to a mean decrease of –28.8 g [95% confidence interval (CI), –49.6 to –8.1], whereas PIAMA’s estimate changed to an apparent increase [47.0 g (95% CI, –10.5 to 104.6)] after controlling for location-specific confounders.

**Figure 4:**
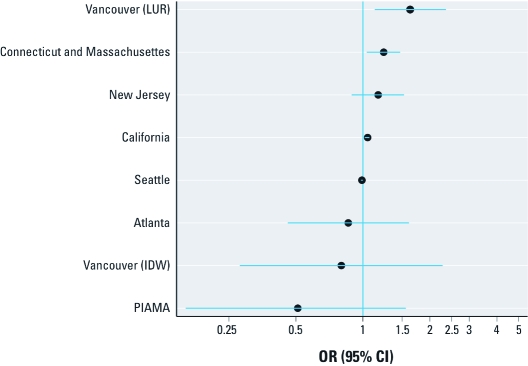
ORs (95% CIs) for LBW among term births in association with a 10‑μg/m^3^ increase in estimated average PM_2.5_ concentration during the entire pregnancy, adjusted for SES, by study. Results for the Vancouver study are from two different PM_2.5_ estimation methods, LUR and IDW of monitor measurements (see "Methods").

Figure 4 shows estimated relative odds of LBW associated with each 10-μg/m^3^ increase in PM_2.5_ concentration, after controlling for SES, for a subset of studies. As for PM_10_, some studies indicated a significant increase in the relative odds of LBW, whereas others indicated no association. The Vancouver study reported different results using different PM_2.5_ estimates. *p*-Values from separate heterogeneity tests, each including one Vancouver estimate, were 0.06 (LUR) and 0.18 (IDW).

## Discussion

Despite the deliberately simple protocol and the heterogeneity in study designs and locations, we found some consistency across studies, particularly for the relationships between PM_10_ and mean birth weight and between PM_2.5_ and LBW. After controlling for SES, the reduction in mean birth weight associated with a PM_10_ increase of 10 μg/m^3^ was between 2 and 20 g for 9 of 11 locations. Although based on fewer studies than those for PM_10_, the initial tests of homogeneity for PM_2.5_ results were not statistically significant. More detailed meta-analysis of the initial results, considering alternative models, influential locations, and differences in location-specific covariates and exposures, may improve our understanding of these relationships and lead to improved summary estimates.

Based on a discussion of initial feasibility study results at the 2009 workshop in Dublin, Ireland (see Appendix), participants concluded that the method used to estimate PM_10_ exposures may be the most critical design difference among the studies. Some prior studies from California (Basu et al. 2004; Wilhelm and Ritz 2005), Vancouver (Brauer et al. 2008), Sydney (Mannes et al. 2005), and Atlanta (Darrow et al. 2009a) have examined the consequences of different methods for calculating pollution metrics in the same study but from different perspectives. For example, as in the results presented in Figure 4, Brauer et al. (2008) compared PM_2.5_ estimates from LUR and monitor data (IDW) and concluded that their moderate correlation could be attributable to different aspects of variability being captured by each method. Basu et al. (2004) found stronger associations for exposures estimated over larger geographic areas than over smaller geographic areas but did not speculate on the reasons for the discrepancy; however, Basu et al. (2004) cautioned that studies using different methods for exposure assessment may not be comparable.

Importantly, there is large variation in PM_10_ levels and concentration ranges among study locations. In the Vancouver study, for example, the 10-μg/m^3^ increase used to derive ORs is nearly an order of magnitude greater than the interquartile range (11.7–13.1; Table 2) of exposures. Similarly, in the Atlanta study, the 10-μg/m^3^ reporting unit represents nearly the entire range of PM_10_ concentrations (18.6–29.6 μg/m^3^).The analytical methods used in the common framework assume no threshold level below which PM is not associated with health. Although evidence supports the hypothesis that no threshold exists for PM relationships and overall population mortality (Daniels et al. 2000), threshold assumptions have not been fully explored for adverse reproductive outcomes, including birth weight. We did not directly examine nonlinear relationships in this feasibility study, but they may contribute to heterogeneity among studies; a more fully coordinated analysis should improve our ability to assess nonlinear relationships.

Covariates likely to affect the relationship between PM_10_ and LBW differ among study locations for many reasons (Strickland et al. 2009). For studies that estimate effects based on spatial contrasts, controlling for SES can be important because it may be spatially correlated with exposure concentrations (O’Neill et al. 2003). However, SES measures and their relationships with both birth outcomes and air pollution are not consistent. For example, although mothers with lower SES generally tend to have poorer birth outcomes, the strength of the relationship differs depending on which birth outcome (birth weight, preterm birth) and which measures of SES (maternal education, occupation) are used (Parker et al. 1994; Pickett et al. 2002). Although in some places mothers with higher SES live in less-polluted areas (Woodruff et al. 2003), in others the opposite relationship holds (Slama et al. 2007). Because participating studies rely on exposure estimates with differing spatial and temporal components, critical confounders may differ among studies (Strickland et al. 2009). Changes between results for the models using SES only and those using SES plus covariates varied among studies, suggesting that other statistical approaches, possibly hierarchical models, that allow for different types of confounding factors could be informative for understanding apparent variations among locations.

Finally, other methods of analysis could be used. Although logistic regression is commonly applied, alternative approaches have considered spatial correlations (Jerrett et al. 2005), time-varying exposures (Suh et al. 2009), generalized additive models (Ballester et al. 2010), and hierarchical structures (Yi et al. 2010). Bell et al. (2007) proposed a method for handling correlated exposures across trimesters. Because both model-based and spatially averaged exposure estimates are calculated with error, considering their precision would provide more accurate confidence intervals (Woodruff et al. 2009).

The ICAPPO feasibility project successfully coordinated analyses of the association between ambient PM concentrations and term LBW, across multiple locations, data sets, and research teams worldwide. These initial results and the participation of multiple research groups, even without external funding, support the continuation of this effort to increase our understanding of the human reproductive consequences of adverse air quality.

## Appendix

We thank Jason Harless for coordinating many aspects of the feasibility study and all of the participants at the 2009 Dublin, Ireland, ICAPPO workshop who contributed their insights and ideas: I. Aguilera, F. Ballester, K. Belanger, M.-H.Chang, G. Collman, M. Dostal, K. Gray, C. Iñiguez, B.-M. Kim, K. Polanska, and J. Rankin.

We thank the principal investigators and scientific teams of the participating centers. For the PIAMA study: B. Brunekreef (Utrecht University and University Medical Center Utrecht, the Netherlands); H.A. Smit [National Institute for Public Health and the Environment (RIVM) and University Medical Center Utrecht, the Netherlands]; A.H. Wijga (RIVM, the Netherlands); J.C. de Jongste (Erasmus University Medical Center/Sophia Children’s Hospital Rotterdam, the Netherlands); J. Gerritsen, D.S. Postma, M. Kerkhof, and G.H. Koppelman (Medical Center Groningen, the Netherlands); and R.C. Aalberse (Sanquin Research, Amsterdam, the Netherlands). The PIAMA study is supported by the Netherlands Organization for Health Research and Development; the Netherlands Organization for Scientific Research; the Netherlands Asthma Fund; the Netherlands Ministry of Spatial Planning, Housing, and the Environment; and the Netherlands Ministry of Health, Welfare, and Sport. For the PAMPER study: L. Parker (Dalhousie University, Halifax, Nova Scotia, Canada) and T. Pless-Mulloli (Newcastle University, Newcastle upon Tyne, United Kingdom). The PAMPER study was supported by the Wellcome Trust (grant No 072465/Z/03/Z). For the Eden study: M.-A. Charles and her group (INSERM 1018 and INSERM–INED joint research team).

For the Vancouver analysis, the linked research database was provided by Population Data BC. Medical services and hospitalization data were provided by the Ministry of Health, Government of British Columbia; Vital Statistics data, by the British Columbia Vital Statistics Agency; and perinatal data, by the British Columbia Reproductive Care Program.

## Supplemental Material

(140 KB) PDF
