# Supplementary material for: The International Collaboration on Air Pollution and Pregnancy Outcomes: Initial Results
Source: Environ Health Perspect. 2011 Feb 9;119(7):1023–8. doi: 10.1289/ehp.1002725 (PMC3222970; doi:10.1289/ehp.1002725)
Supplement: (140 KB) PDF [file ehp.1002725.s001.pdf]

The International Collaboration on Air Pollution and Pregnancy Outcomes: Initial Results.

Supplemental Material

Jennifer Parker, David Q. Rich, Svetlana V. Glinianaia, Jong Han Leem, Daniel Wartenberg, Michelle L. Bell, Matteo Bonzini, Michael Brauer, Lyndsey Darrow, Ulrike Gehring, Nelson Gouveia, Paolo Grillo, Eunhee Ha, Edith H. van den Hooven, Bin Jalaludin, Bill M. Jesdale, Johanna Lepeule, Rachel Morello-Frosch, Geoffrey G. Morgan, Rémy Slama, Frank H. Pierik, Angela Cecilia Pesatori, Sheela Sathyanarayana, Juhee Seo, Matthew Strickland, Lillian Tamburic, and Tracey J. Woodruff

Table 1: Additional covariates included in study-specific models (Model 2) as reported by study.

Study	Additional covariates
Atlanta	Indicator variables for year of LMP date and month of LMP date, race/ethnicity, marital status, parity (primi vs >1), maternal age (<20, 20-34, >34), maternal smoking (yes/no/missing), gender, gestational age (class variable), maternal education (<12 vs 12+ years)
California	Maternal age, race/ethnicity, marital status, prenatal care, maternal risk factors, neighborhood socioeconomic characteristics
Connecticut and Massachusetts	Gestation categorized by weeks, mother's age (<20,20-24,25-29,30-34,35-39,40+), 1st baby (yes/no), mother's race (Black/White/Other), mother smoked during pregnancy (yes/no/missing)
EDEN	Gestational duration (continuous), gestational duration² (continuous), maternal age at conception (continuous), maternal age at conception² (continuous), parity status (nulliparous, multiparous), gender (male, female)
Lombardy	Maternal nationality (5 categories), mode of delivery (6 categories), paternal job (6 categories), maternal age (continuous), gender (females vs males), parity (nulliparous vs. multiparous), gestational duration (continuous within 37-42 weeks)
PAMPER	Gestational age coded as single weeks 37-42 (continuous), parity, maternal age in years (continuous)
New Jersey	Maternal age, race, marital status, trimester of prenatal care initiation, smoking, drinking and drug use during pregnancy
PIAMA	Maternal smoking during pregnancy, gender, older siblings (yes no), gestational age
Generation R	Maternal age (continuous), gestational age (37-42 wk), gender, parity (nulliparous/multiparous/missing), maternal ethnicity (5 groups), maternal smoking (yes/no/missing)
São Paulo	Maternal age, parity, antenatal care visits

Seattle	Marital status(0/1), income categories(0-3), gestational age, baby sex, maternal age, race, smoking status and parity
Seoul	Maternal education(5 categories), gestational age, baby sex, maternal age
Sydney	Maternal age (<20 yrs, 20-24 yrs, 25-29 yrs, 30-34 yrs, 35+ yrs), indigenous status (0/1), country of birth (0/1/2), gender, maternal smoking (0/1), parity, maternal hypertension (0/1), maternal gestational hypertension (0/1), maternal diabetes (0/1), maternal gestational diabetes (0/1), antenatal care before 12 weeks (0/1), socio-economic disadvantage (quartiles), year of birth, season of birth
Vancouver	Gender (M/F), mother's smoking (Y/N), birth year, birth month, income quintile for census area (1=poorest), parity (multiparous vs not), female education (quartiles of % women in census area with post-secondary education), maternal age(1:0-19;2:20-29;3:30-34;4:35-39;5:ge 40), first nations status (Y/N)

Table 2: Odds ratios (and 95% confidence intervals) for low birth weight among term births in association with a 10 $\mu g/m^3$ increase in estimated average PM_{10}, or black smoke (PAMPER), concentration during the entire pregnancy, adjusted for socioeconomic status (SES) (Figure 1)and both SES and location-specific variables (Figure 2), by study.

Study	OR (95% Confidence Interval)			
	Adjusted for SES		Adjusted for SES and study-specific covariate	
Atlanta	1.11	1.01 to 1.22	0.96	0.71 to 1.29
California	1.01	1.01 to 1.02	1.01	1.00 to 1.02
Connecticut and Massachusetts	1.13	1.06 to 1.20	1.08	1.02 to 1.15
EDEN	0.97	0.38 to 2.49	1.05	0.39 to 2.85
Lombardy	0.98	0.95 to 1.01	0.99	0.96 to 1.02
PAMPER	1.02	1.01 to 1.02	1.01	1 to 1.02
New Jersey	0.92	0.85 to 1.01	1.00	0.91 to 1.09
PIAMA	0.63	0.3 to 1.35	0.81	0.24 to 2.76
Generation R	0.89	0.21 to 3.73	1.11	0.25 to 4.87
São Paulo	1.13	1 to 1.27	1.15	1.02 to 1.29
Seoul	1.10	(1.06 to 1.14)	1.05	(1.01 to 1.09)
Sydney	1.10	(0.98 to 1.23)	1.22	(1.09 to 1.37)
Vancouver	1.15	(0.61 to 2.18)	1.44	(0.62 to 3.36)

Table 3: Change in mean birth weight, g, (and 95% confidence intervals) among term births in association with a 10 μg/m^3 increase in estimated average PM_{10}, or black smoke (PAMPER), concentration during the entire pregnancy, adjusted for socioeconomic status (SES) (shown in Figure 3) and for both SES and study-specific covariates, by study.

Study	Beta (95% Confidence Interval), grams	
	Adjusted for SES	Adjusted for SES and study-specific covariates
Atlanta	21.0 (13.6 to 28.3)	-28.8 (-49.6 to-8.1)
California	-8.1 (-8.8 to -7.5)	-11.1 (-11.8 to -10.4)
EDEN	-15.1 (-73.9 to 43.8)	-16.3 (-72.2 to 39.6)
Lombardy	8.9 (6.6 to 11.3)	6.4 (4.3 to 8.5)
PAMPER	-2.0 (-2.5 to -1.6)	-4.7 (-5.2 to -4.3)
PIAMA	-20.5 (58.6 to17.5)	47.0 (-10.5 to104.6).
Generation R	-42.2 (-147.3 to 62.9)	-10.3 (-102.9 to 82.2)
São Paulo	-15.6 (-25.3 to -5.9)	-18.3 (-28.5 to -8.1)
Seoul	-2.2 (-4.1 to -0.3)	Not available
Sydney	-19.9 (-26.6 to -13.2)	-30.3 (-36.4 to -24.2)
Vancouver	-7.0 (-10.5 to -3.6)	-7.2 (-11.7 to -2.7)
